# Automatic lung segmentation of magnetic resonance images: A new approach applied to healthy volunteers undergoing enhanced Deep-Inspiration-Breath-Hold for motion-mitigated 4D proton therapy of lung tumors

**DOI:** 10.1016/j.phro.2024.100531

**Published:** 2024-01-04

**Authors:** John H. Missimer, Frank Emert, Antony J. Lomax, Damien C. Weber

**Affiliations:** a Center for Proton Therapy, Paul Scherrer Institute, Villigen PSI, Switzerland; b Department of Physics, ETH Zurich, Zurich, Switzerland; c Department of Radiation Oncology, University Hospital Zurich, Zurich, Switzerland; d Department of Radiation Oncology, Inselspital, Bern University Hospital, University of Bern, Switzerland

**Keywords:** MRI, lung volume, Automated segmentation, Motion mitigation, Enhanced DIBH, Proton therapy

## Abstract

**Background and purpose:**

Respiratory suppression techniques represent an effective motion mitigation strategy for 4D-irradiation of lung tumors with protons. A magnetic resonance imaging (MRI)-based study applied and analyzed methods for this purpose, including enhanced Deep-Inspiration-Breath-Hold (eDIBH). Twenty-one healthy volunteers (41–58 years) underwent thoracic MR scans in four imaging sessions containing two eDIBH-guided MRIs per session to simulate motion-dependent irradiation conditions. The automated MRI segmentation algorithm presented here was critical in determining the lung volumes (LVs) achieved during eDIBH.

**Materials and methods:**

The study included 168 MRIs acquired under eDIBH conditions. The lung segmentation algorithm consisted of four analysis steps: (i) image preprocessing, (ii) MRI histogram analysis with thresholding, (iii) automatic segmentation, (iv) 3D-clustering. To validate the algorithm, 46 eDIBH-MRIs were manually contoured. Sørensen-Dice similarity coefficients (DSCs) and relative deviations of LVs were determined as similarity measures. Assessment of intrasessional and intersessional LV variations and their differences provided estimates of statistical and systematic errors.

**Results:**

Lung segmentation time for 100 2D-MRI planes was ∼ 10 s. Compared to manual lung contouring, the median DSC was 0.94 with a lower 95 % confidence level (CL) of 0.92. The relative volume deviations yielded a median value of 0.059 and 95 % CLs of −0.013 and 0.13. Artifact-based volume errors, mainly of the trachea, were estimated. Estimated statistical and systematic errors ranged between 6 and 8 %.

**Conclusions:**

The presented analytical algorithm is fast, precise, and readily available. The results are comparable to time-consuming, manual segmentations and other automatic segmentation approaches. Post-processing to remove image artifacts is under development.

## Highlights

• A new magnetic resonance imaging (MRI) lung segmentation algorithm was developed.
• Validated on manual MRI lung contours it automatically produces comparable results.
• Compared against other MRI algorithms it is fast, precise, and easy-to-implement.
• Its lung volume determination could optimize motion-mitigated 4D proton therapy.
• Successful usage was shown in a trial on breath-hold for respiratory suppression.

## 1 Introduction

Pencil-beam-scanning proton therapy (PBS-PT) is gaining attention in radiation oncology for its potential and application in the treatment of lung tumors [1], [2], [3]. To improve treatment outcomes, 4D proton irradiation with motion mitigation strategies has been and continues to be explored [4], [5], [6]. Magnetic resonance imaging (MRI) has emerged as a valuable radiation-free tool for studying lung function and respiratory processes [7], [8], [9].

While 4D-MRIs play an important role in the analysis, modeling and image guidance of dynamic respiratory motion [10], [11], [12], [13], [14], motion artifacts in MRI lung reconstructions can be critical [15]. Although artificial intelligence has advanced motion compensation [16], a preferred method for limiting image artifacts is MRI acquisition under breath-hold conditions, where “oxygen administration and patient coaching […] can increase breath-hold capability” [17].

At the same time, promising respiratory motion management strategies exist [18], [19], [20], such as enhanced Deep-Inspiration-Breath-Hold (eDIBH), which would allow active suppression of respiratory motion and prolonged breath-hold during PBS-PT [21], [22]. In an MRI-based study, we demonstrated the feasibility of this approach by simulating and recording quasi-static PBS-PT lung irradiation fractions in an MR scanner in healthy volunteers [21]. Such quasi-steady-state conditions would allow the well-known physical advantages of proton therapy in the treatment of stationary tumors [23], [24], [25], [26] to be directly applied to the irradiation of lung tumors [27], [28], at least for a representative duration of a field application [29]. In contrast, time-dependent 4D-PBS-PT in lung patients is much more complex and partially requires considerable technical effort [30], [31], [32], e.g., for beam tracking [33].

To spare healthy lung tissue and to minimize key dosimetric lung irradiation parameters (D_mean_, V20%), e.g., to reduce the risk for radiation pneumonitis, it is crucial to achieve the largest possible lung volume (LV), i.e., to maximize it, ideally by reaching total lung capacity (TLC) at full inspiration [34]. This is critical for both irradiation [35], [36] and computed tomography (CT) simulation for treatment planning [37]. Our MRI-based study has shown that TLC conditions under eDIBH provide excellent reproducibility of lung topology, including potential tumor locations and adjacent structures [21]. Accurate LV determination by reliable, effective contouring is paramount to accurately quantify e.g., D_mean_ and V20% [38]. For its use in online-adaptive, conventional radiotherapy (RT) under image guidance, such as MR-guided RT (MRgRT), computational speed could also be crucial [39]. While CT-based 4D-PBS-PT planning benefits from an extensive software toolbox for automated lung contouring and volume determination, the availability of such programs for MRI-based segmentation is extremely limited.

Several algorithms have been proposed for accurate lung segmentation using MRI data, some of which rival manual segmentation by experts. These algorithms may use morphological filtering, region growing [40], normalization with an atlas library of lung segmentations [41], or artificial neural networks [42], [43], [44]. However, most of these methods require 3D-MRI acquisitions.

The objective of this study was to develop, apply and test an automated 2D-MRI algorithm for lung segmentation with simultaneous volume determination based on a commercial scientific image processing platform [21]. Its accuracy, reliability, and speed were investigated in the context of MRI-assisted simulations of lung tumor treatments with protons applying eDIBH. For validation, the results of LV determinations were compared to manual segmentations and quantified using established similarity metrics such as DSCs [45] and fractional volume deviations. The algorithm is intended to provide an alternative to existing methods which often rely on more complex 3D-MRIs or artificial intelligence (AI)-based approaches.

## 2 Materials and methods

### 2.1 Subject population

A total of 21 healthy subjects, 9 females and 12 males (40–58 years; body mass index (BMI) 19–29.6 kg/m2), representing a potential lung cancer patient cohort, participated in the described eDIBH part of the study protocol. Prior to each MRI session, lung function was assessed by forced spirometry; the subjects presented forced vital capacity (FVC) between 3.25L and 7.26L (Table S1). The study was approved by the Cantonal Ethics Committee Northwest/Central Switzerland (BASEC-ID: 2018–01295; clinicaltrials.gov: NCT03669341). Subjects were thoroughly informed of the study objectives and procedures and signed an informed consent form.

### 2.2 MRI data acquisition

Subjects in our study were immobilized on the MR couch in a head-first-supine position with arms overhead. MRI data were acquired using a T2-weighted, 2D-SSFP (steady-state free-precession) sequence on a 1.5 T MR scanner (MAGNETOM Aera, Siemens Healthcare AG, Erlangen, Germany). Voxel spacing was 0.7617x0.7617 mm^2^ in-plane, with a plane separation of 2.2 mm and a slice thickness of 5.5 mm. The reconstructed image was a 512x512 pixel array with pixel intensities stored as 16-bit unsigned integers ranging from 400 to 1800 (arbitrary units). A single MRI of the entire lung, stored in DICOM format, consisted of ∼ 100 coronal 2D image slices acquired in ∼ 70 s.

Each participant underwent four MRI sessions at weekly intervals over a three-week period, each session containing two consecutive MRI acquisitions under eDIBH. eDIBH consisted of breath-holding under full inspiration during image acquisition, preceded by an 8-min period of 100 %–O2 breathing with final hyperventilation. Before each MRI, eDIBH was restarted. The subject remained in the same position between both scans to simulate intrafractional conditions similar to those between two radiation fields. A total of 168 eDIBH-MRIs were acquired.

### 2.3 Automated segmentation algorithm

**Fig. 1:**
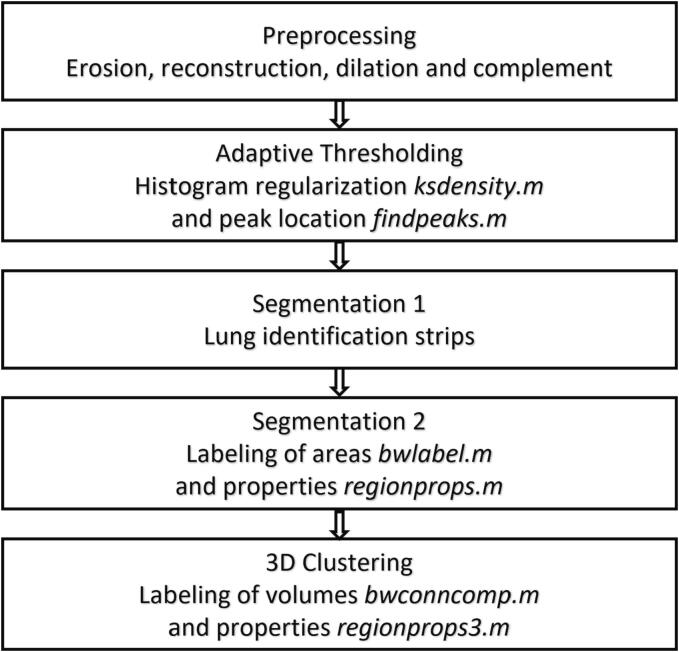
Workflow diagram of segmentation algorithm.

**Fig. 2:**
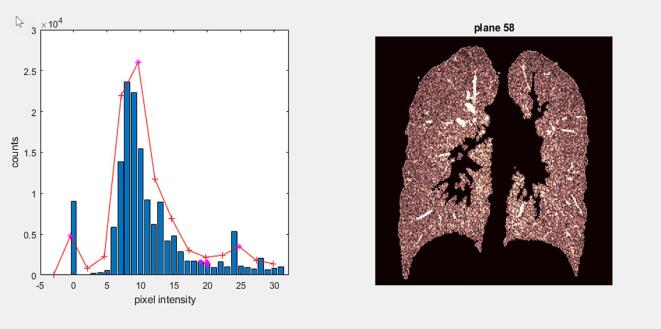
Example of adaptive thresholding for plane 58 of a sample acquisition In the left panel the blue bars indicate the distribution of pixel intensities between 0 and 32 as shown on the horizontal axis. The red line denotes the kernel smoothing with the magenta stars (*) indicating the peaks of the smoothed distribution. The magenta diamond (◊) at pixel intensity 20 demarks the local minimum; the magenta circle (○) the adjusted threshold. The right panel shows the segmented lung. (For interpretation of the references to colour in this figure legend, the reader is referred to the web version of this article.)

The segmentation algorithm was implemented using Matlab™ (The MathWorks, Inc., Natick, Massachusetts, United States), by directly importing DICOM files. It included four procedures: (i) image preprocessing, (ii) MRI histogram analysis with thresholding, (iii) automatic segmentation, (iv) 3D-clustering (Fig. 1). To develop and validate the algorithm, adaptive thresholding, lung identification and characterization were presented plane-by-plane in an additional graphical display (Fig. 2), with all MRIs evaluated at least once.

Preprocessing included the morphological transformations erosion, reconstruction, dilation, and complement [46], which were applied with a 12-pixel disk for each MRI plane, preserving anatomical structures and equalizing pixel values within regions [47] (Fig. S1).

Adaptive thresholding determined the segmentation threshold for each plane. The graphical display indicated that the MRI acquisition protocol resulted in pixel intensity distributions with maxima below 12 intensity units per pixel. The spectrum of possible pixel intensities showed a maximum of 31 intensity units, suggesting a maximum threshold of 32 for lung segmentation. Kernel smoothing with a Gaussian kernel and a bandwidth of one pixel intensity unit was performed to regulate fluctuations in the histograms using function *ksdensity.m*. The maximum of the distribution was found with the function *findpeaks.m* (Fig. 2).

The segmentation threshold was determined as the minimum to the right of the maximum and less than one-tenth of the maximum. In cases where no such minimum existed, the threshold was chosen as the minimum between the global maximum (left) and the next highest maximum (right) beyond 12 pixel intensity units. If no next higher local maximum existed, the threshold closest to the global maximum was accepted and recorded as an exception.

For consistency, the estimated threshold for each plane, except the first, was compared to that of the previous plane. If the difference exceeded two pixel intensity units, the current threshold was adjusted accordingly, assuming similar distributions for adjacent planes.

Regions of the plane with pixel values below the threshold yielded binary masks that could be isolated or connected to the image boundary. To verify that the masked regions were lung and account for occasional disconnections between contiguous lung segments on one side, bilateral test strips were defined on all 2D planes. The presumed lung segments had to intersect the test strips. The inner test strip boundaries were set 76 pixels to the left and 99 pixels to the right of the center, each 21 pixels wide, and extending from 116 pixels below to 124 pixels above the midplane (Fig. S2). The graphical display was used to determine appropriate strip dimensions for the subject cohort.

Regions containing the plane boundary were decomposed into the periphery of vanishing pixel values defined by Matlab™ and disconnected regions. The functions *bwlabel.m* and *regionprops.m* enumerated disconnected and isolated regions and determined their respective sizes. These regions were then sorted by size and examined for intersection with the test strips. The regions overlapping the test strips formed the segmented masked plane.

3D-clustering was performed using *bwconncomp.m* to label connected components in the volume of the segmented binary masks. The volumes of these components were determined using *regionprops3.m* and sorted by size. Components intersecting the test strips were considered part of the lung. Flipping the second largest component around the vertical axis and calculating its intersection with the largest volume determined whether the volume comprised one or two lobes, thus excluding numerous residual volumes. The segmented images derived from the binary masks were saved in DICOM format, while LVs, thresholds, and exceptions were saved in Matlab™ format.

The algorithm could not distinguish between lung and parts of the trachea and certain protuberances. To quantify these artifacts, Velocity™, a certified oncology imaging informatics system (Varian Medical Systems, Palo Alto, USA) (https://www.varian.com/oncology/products/software/velocity) was used to contour them using the functionality *paint* and determine their volumes.

### 2.4 Reproducibility and variability

In addition to validation using the graphical display, the automated segmentation algorithm was further validated by manual segmentation of 46 out of the 168 eDIBH-MRIs. Each subject contributed at least one comparison, most at least two, except one subject with four comparisons. Two medical assistants performed the contouring using Velocity™ under the supervision of an experienced radiation oncologist. The algorithm segmentations were then compared to the manual segmentations using the DSCs. Fractional deviations between the respective lung volumes (VFD = 2*(V_man_-V_auto_)/(V_man_ + V_auto_)) served as an additional similarity measure.

Pairs of subsequent MRIs within a session repeated in all four sessions, provided insight into the reproducibility of lung volume determinations, with intrafractional and interfractional LV variations providing rough limits of uncertainty. As measure of between-session variation, the SD of the four session means was calculated for each subject, yielding 21 SDs. This fractional SD was calculated as the ratio of SD to mean deviation.

## 3 Results

### 3.1 Adaptive Thresholding and Segmentation

**Fig. 3:**
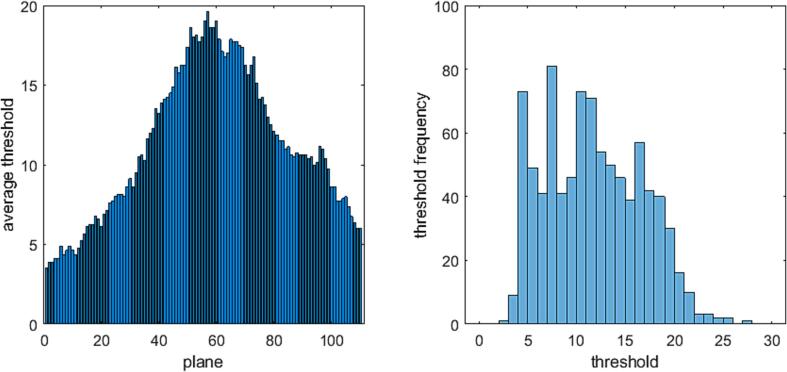
Distribution of average thresholds and related histogram for one subject (8 MRIs). The left panel displays the distribution per plane of average thresholds in pixel intensity units for the eight acquisitions of one subject. The right panel shows the frequency distribution of thresholds with respect to pixel intensity units.

The threshold dependence on plane for a single subject was illustrated in Fig. 3

The eight segmentations computed for the subject yielded the average per plane. Typical for all segmentations were the low thresholds (∼5) for the first planes, the highest thresholds (∼20) for the middle planes and a shoulder at (∼10) around plane 80. The right panel showed the distribution of thresholds for the 8 segmentations. For almost all images the maximum frequencies occurred between 10 and 15-pixel intensity units (gray values); excursions beyond 25 units were rare. These plots confirmed the choice of 12 units as the critical intensity for determining the threshold and 32 units as the maximum search range. The number of exceptions, i.e., planes in which the threshold was taken as the minimum closest the distribution maximum regardless of amplitude, ranged from 2 to 4 for the 8 segmentations, a comparatively low value. In a subset of 15 subjects, the number of exceptions in a segmentation ranged from zero to 78 with median 15 and peak maximum at 2.

In two of the 168 MRIs, segmentations from one subject in one session showed algorithmic failure by visual inspection; two segmentations from the same subject in a subsequent session appeared to be accurate. The MRI acquisition log files of the failed segmentation showed no obvious anomalies.

Calculation time for an MRI segmentation was about 10 s on a HP® Z2 tower G5 workstation with processor intel Core®i7-10700-CPU@2.90 GHz

### 3.2 Comparison of Algorithm with Manual Segmentation

**Fig. 4:**
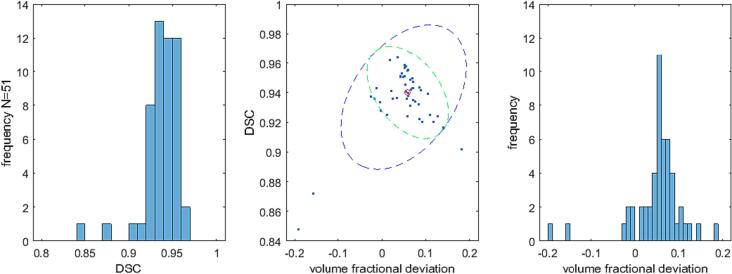
Distributions of DICE similarity coefficients (DSC) of and fractional volume deviations for comparison of manual and automated segmentation. Indicated in the middle panel are the 95%-CL boundaries for the joint distributions with (blue dashed) and without (cyan dashed) the two outliers. The red circle denotes the mean of the distribution excluding the two outliers. The left and right panels show individual distributions of DSCs and volume fractional deviations, respectively. (For interpretation of the references to colour in this figure legend, the reader is referred to the web version of this article.)

All DSCs, except for two outliers, exceeded 0.9 (Fig. 4), median 0.94, 95 %-CL [0.92, 0.97]. Volume fractional deviations were predominantly positive, exceeding −0.024, median 0.059, 95 %-CL [-0.013, 0.13]. Manual segmentation often yielded slightly larger LVs.

**Fig. 5:**
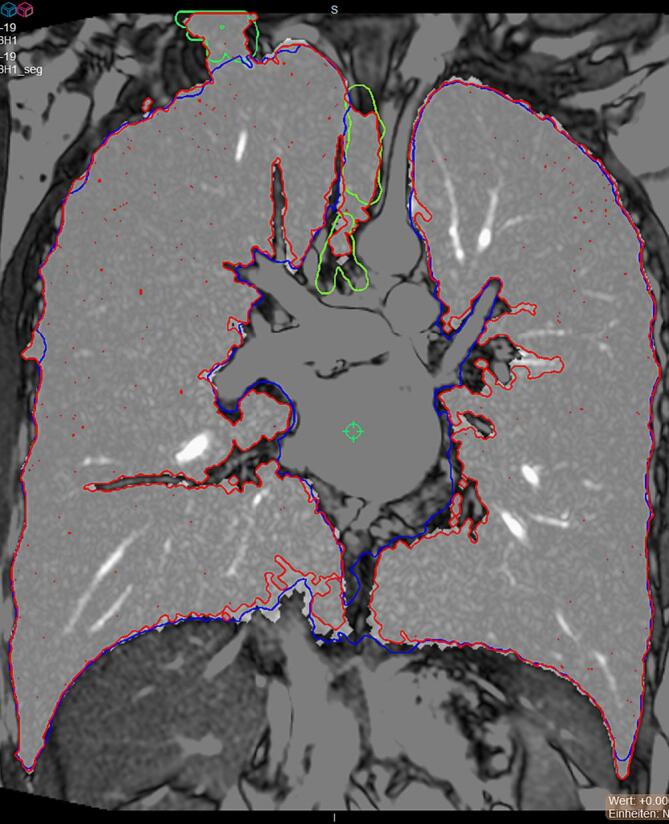
Comparison of segmentation methods and illustration of artifacts. In the displayed acquisition of one subject red delineates automatic segmentation, blue manual segmentation. Green delineates artifacts: generated in the trachea and on the superior lobe of the right lung. (For interpretation of the references to colour in this figure legend, the reader is referred to the web version of this article.)

Visual inspection of the segmentation methods revealed: (i) manual segmentation provided good outer contours but did not account well for blood vessels and bronchi in inner contours and (ii) the algorithm occasionally produced protuberances from the outer contours, did not distinguish the trachea nor separate connected right and left lung segments. However, internal blood vessels and bronchi were often better represented than in manual segmentation. Visual inspection showed that manual segmentation overestimated volumes. The algorithm showed massive protuberances extending from the outer contours in the computed segmentations of the two outliers while contours for at least one plane were missing in the manual segmentations. Fig. 5 showed a sample comparison for a single plane.

### 3.3 Reproducibility, Variability and Image Artifacts

Table S2 lists the LVs measured in all eDIBH-MRIs. Four pairs of eDIBH-MRIs performed within sessions for 21 subjects yielded 4 intrafractional deviations from each of the 21 subject means. The mean of these volume fractional deviations was 0.0167 with standard deviation (SD) 0.058; 68 of the 84 eDIBH-MRI pairs (81 %) were within one SD (Fig. S3). 17 of 21 determinations yielded variations of less than 7 % with a median of 3 % (Fig. S4).

**Fig. 6:**
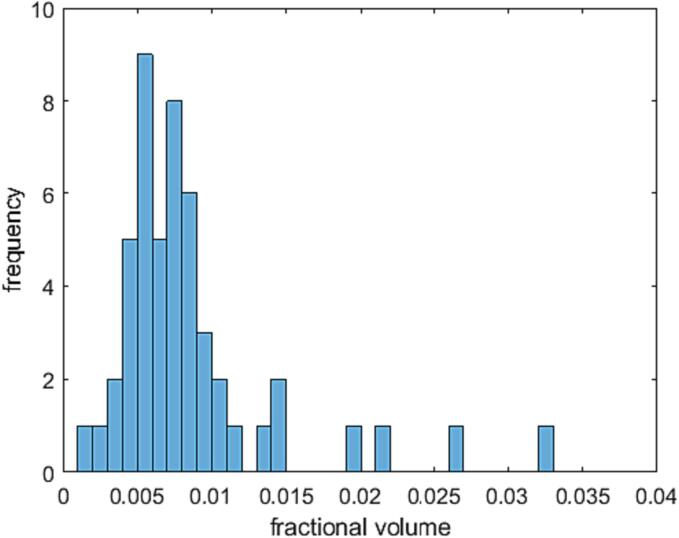
Total fractional volumes of artifacts.

The total fractional volume of artifacts, i.e., the ratio of artifact to total lung volume, ranged up to 3.2 % with a median of 0.7 % and, excluding outliers, less than 2.2 % with 95 %-CL (Fig. 6). Fig. S5 showed that the trachea was the dominant artifact. In 8 of 46 volumes evaluated, it was the only artifact; in 34 of 46 volumes, it contributed more than 50 % of the artifact volume.

Artifacts were evaluated for 51 lung volumes, including the 46 selected for manual segmentation. They occurred almost exclusively in the mid-planes, where lung is largest. The lung was sometimes connected to the image boundaries by thin fibrils; trachea and protrusions were also evident.

Volume fractional deviations reflected systemic as well as statistical deviations. Image artifacts were an important source of systematic deviations

## 4 Discussion

We presented an adaptive threshold-based lung segmentation algorithm using standard Matlab™ modules for morphological transformations, regional classification, and 3D- clustering. Developed in the context of respiratory suppression motion mitigation methods for 4D proton therapy, the algorithm was applied after 2D-MRI acquisition under eDIBH conditions, providing accurate and reproducible measurements of total LVs within seconds. The short MRI acquisition time (∼1min) was comparable to the time required to deliver an average proton fraction field at PSI's Gantry-2 for a typical lung tumor [21] and could be achieved by lung patients practicing a variant of eDIBH [29].

The study cohort consisted of healthy middle-aged women and men with representative weight and height distributions. Analysis of the MRIs provided empirically derived lung descriptors in the form of rectangular test strips to facilitate the identification of isolated lung segments. We would expect them to be suitable for an adult cohort with much greater age variation, but they could easily be adapted for other cohorts, such as children.

Accuracy and reproducibility were assessed by an evaluation of intra- and interfractional LV variability. Intrafractional variability was below 3 % (68 out of 84 eDIBH-MRI pairs), while interfractional variability was less than 4 % in 14 of 21 subjects. These variations compared well with the intersession variability of FVC measurements (2 %) obtained by spirometry [21], [46].

Comparing manual and automatic segmentation, all DSCs exceeded 0.9, with a median of 0.94 and a 95 % confidence interval (CI) [0.92 0.98], except for two outliers. Additionally, the VFD showed a median of 0.059 with a 95 %-CI [-0.01, 0.13]. Predominantly positive VFD values indicated that manual segmentation typically yielded slightly larger LVs than the algorithm. Manual segmentation, unlike automatic segmentation, often included vessels and airways in the anterior lung (Fig. 5).

Our DSC and VFD values compared favorably with those reported by [40], who obtained DSCs of 0.93 ± 0.01 for the left lung and 0.94 ± 0.01 for the right lung when comparing their algorithm with manual segmentations by experienced chest radiologists. These values were slightly lower than those reported by [41], who used an atlas-based method with expert manual segmentations performed in two steps, yielding DSCs of 0.981 ± 0.007 for the left lung and 0.984 ± 0.006 for the right lung. The DSCs of neural network segmentations applied to 3D-MRI acquisitions using fast ultra-short echo time sequences were higher than those of our algorithm. DSCs of 0.97 with a 95 %-CI [0.96, 0.97] for the right lung and 0.96 with a 95 %-CI of [0.96, 0.97] for the left lung were found in [42] using manual segmentations complemented by the region growing algorithm of [38] as reference. In [44] a total DSC of 0.967 ± 0.076 was observed for lung tissue when using manual segmentation by an experienced radiologist as a reference.

Analysis of artifacts suggested that our algorithm might overestimate LVs by up to 2 % (Fig. 5). These artifacts were most pronounced in mid-planes, where thresholds were highest, with the trachea contributing the most. Removal of the artifacts could reduce volume fractional deviations and potentially increase DSCs by at least 0.01. Nevertheless, the LV errors due to image artifacts were smaller than inter- and intrasession deviations.

In terms of computational time, our algorithm required approximately 10 s to segment an LV on 100 2D slices. This time was comparable to the 0.087 s per 2D slice plane reported by [44], favorable compared to the 1 min reported by [40] and [41], and 46 s reported by [42].

In terms of limitations, testing the algorithm with other MR protocols or scanner models was hampered by resource constraints. However, the flexibility of adaptive thresholding suggested potential application with parameter adaptations to different 2D-MR acquisition protocols assuming adequate lung contrast. Pending post-processing might remove artifacts and separate lung lobes.

When evaluating lung segmentation, both volume-based metrics such as DSC and distance-based metrics such as Hausdorff distance were typically used for a thorough comparison (e.g. [47]). In scenarios where the DSC values were in the range of 0.95, indicating a high degree of volume overlap, the distance-based metrics typically provided distances of a few mm which only slightly affects the resulting LV [48]. As our primary goal was to determine the accuracy of TLC determination, which is only minimally affected by superficial and internal lung structures, we did not specifically assess Hausdorff distance in our analysis.

Although tested exclusively in healthy subjects, experience with other algorithms [40], [41] suggested potential utility for lung tumor patients in conjunction with eDIBH. Despite these limitations, our algorithm provided analytical, easily-available, fast and accurate automatic segmentation for 2D-MRIs using standard Matlab™ features. This could significantly contribute to 4D proton therapy with motion mitigation techniques such as enhanced DIBH and MR-based radiation oncology imaging, supporting the optimization of dosimetric irradiation parameters for improved therapeutic outcomes in lung patients.

## 5 Code availability statement

Matlab™ code supporting the conclusions of this article will be provided by the authors upon reasonable request.

## Declaration of competing interest

The authors declare that they have no known competing financial interests or personal relationships that could have appeared to influence the work reported in this paper.
