# Supplementary material for: Automatic lung segmentation of magnetic resonance images: A new approach applied to healthy volunteers undergoing enhanced Deep-Inspiration-Breath-Hold for motion-mitigated 4D proton therapy of lung tumors
Source: Phys Imaging Radiat Oncol. 2024 Jan 4;29:100531. doi: 10.1016/j.phro.2024.100531 (PMC10825631; doi:10.1016/j.phro.2024.100531)
Supplement: Supplementary data 1 [file mmc1.docx]

**Supplementary Material**

|  | Women (9) | Men (12) |
| --- | --- | --- |
|  | min med max | min med max |
| Age [year] | 40 48 53 | 43 49 58 |
| Weight [kg] | 50 62 74 | 68 90 100 |
| Height [cm! | 158 169 177 | 173 185 190 |
| BMI [kg/m^2^] | 19.0 22.8 25.2 | 22.5 26.9 29.6 |
| FVC [L] | 3.25 3.98 4.31 | 4.24 5.78 7.26 |

Table S1: Subject demographics
*(BMI: Body Mass Index; FVC: Forced Vital Capacity)*


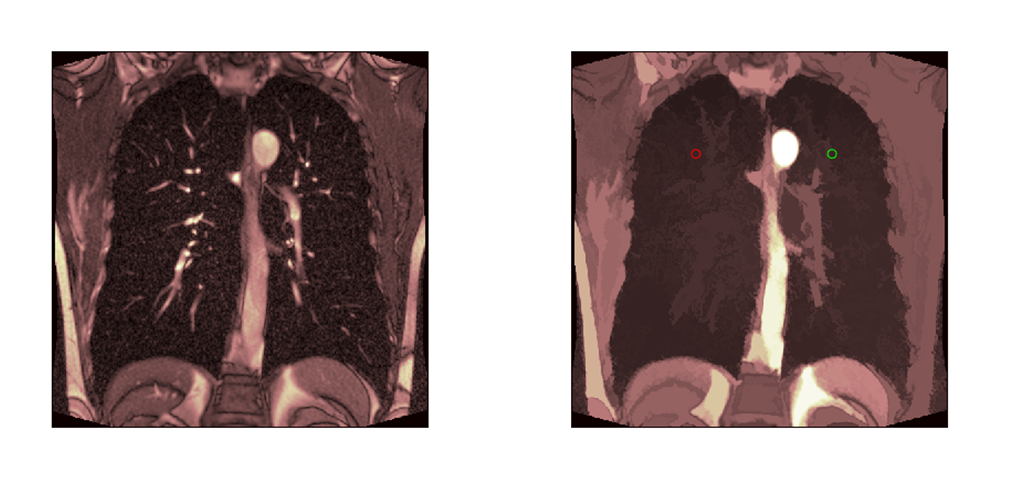


Figure S1: Example of preprocessing

*The left panel displays the raw image whereas in the right panel the result of the morphological transformation is shown.*


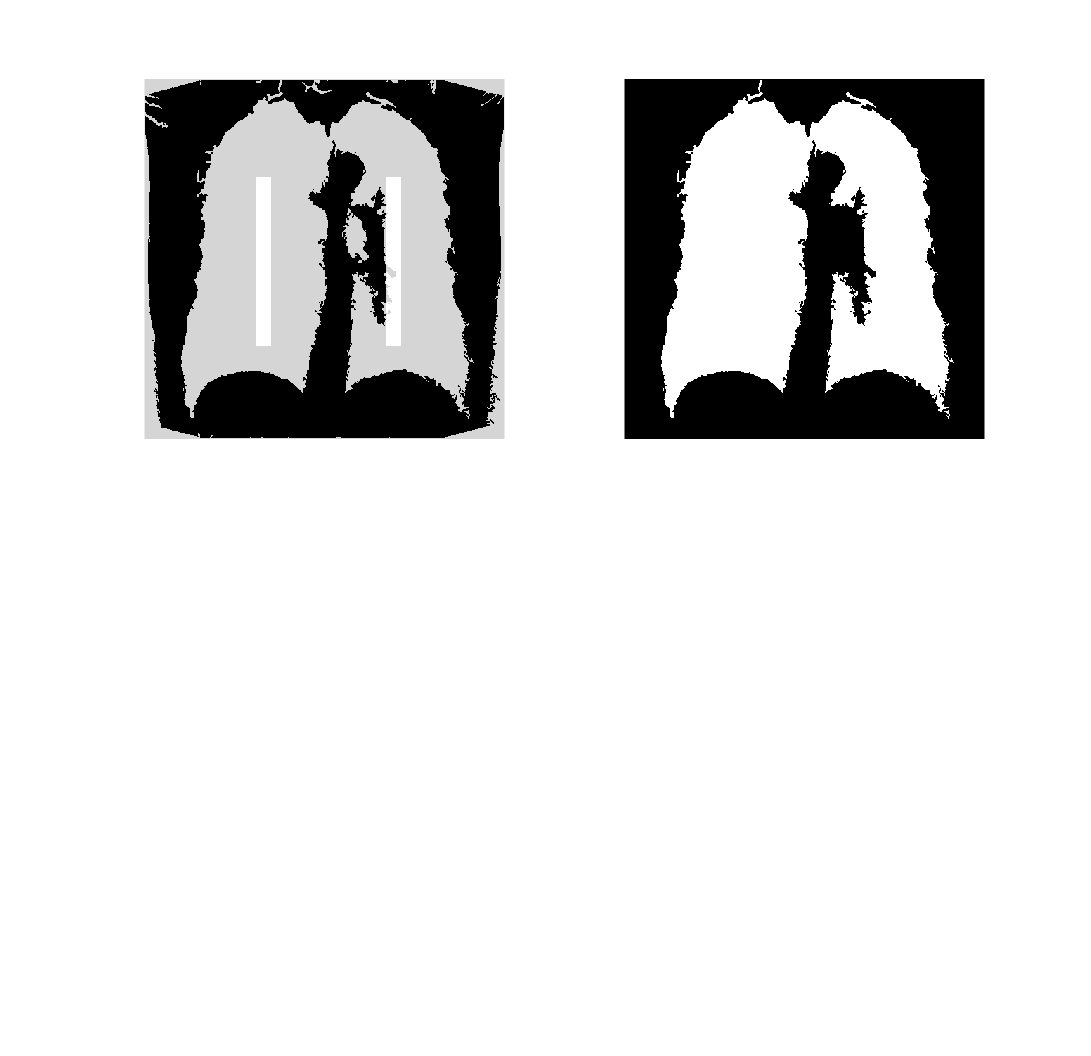


Figure S2: Example of segmentation procedure using test strips

*Taking the first of the 8 eDIBH-MRI scans of subject 5 (plane 63) as an example, the left panel shows the test strips superimposed on the proper boundary determined by Matlab™. The lung segments are connected to one another and to the boundary by thin tendrils. The right panel displays the segmented lung after characterization and checking for intersection with the test strips.*

| Subject / Session | S1_1 | S1_2 | S2_1 | S2_2 | S3_1 | S3_2 | S4_1 | S4_2 |
| --- | --- | --- | --- | --- | --- | --- | --- | --- |
| 1 | 5.93 | 5.74 | 5.80 | 5.75 | 6.01 | 5.96 | 6.08 | 6.30 |
| 2 | 5.09 | 4.86 | 5.01 | 4.88 | 4.82 | 4.91 | 5.03 | 4.95 |
| 3 | 7.53 | 7.46 | 7.64 | 7.49 | 7.53 | 7.42 | 7.48 | 7.72 |
| 4 | 6.51 | 6.61 | 6.68 | 6.50 | 6.44 | 6.57 | 6.38 | 6.37 |
| 5 | 7.94 | 7.73 | 7.88 | 8.01 | 7.91 | 7.77 | 7.83 | 7.27 |
| 6 | 5.61 | 4.99 | 5.72 | 5.86 | 5.69 | 5.48 | 5.64 | 5.47 |
| 7 | 6.15 | 6.11 | 6.41 | 6.25 | 6.55 | 6.39 | 6.27 | 6.10 |
| 8 | 7.72 | 7.65 | 7.51 | 6.94 | 7.53 | 7.52 | 7.52 | 7.43 |
| 9 | 4.56 | 4.51 | 4.48 | 4.42 | 4.59 | 4.14 | 4.38 | 4.42 |
| 10 | 7.15 | 7.06 | 7.03 | 7.06 | 7.09 | 7.07 | 6.93 | 6.94 |
| 11 | 4.80 | 4.65 | 4.32 | 3.08 | 3.74 | 3.16 | 3.71 | 4.18 |
| 12 | 4.06 | 3.77 | 4.85 | 4.99 | 5.17 | 4.93 | 5.01 | 5.09 |
| 13 | 4.51 | 4.40 | 4.69 | 4.51 | 4.74 | 4.93 | 4.75 | 4.67 |
| 14 | 4.40 | 4.44 | 4.42 | 4.52 | 4.27 | 4.26 | 4.33 | 4.19 |
| 15 | 5.00 | 5.07 | 5.20 | 4.88 | 5.58 | 5.72 | 5.86 | 5.47 |
| 16 | 5.28 | 5.33 | 5.58 | 5.48 | 5.28 | 5.36 | 4.53 | 5.11 |
| 17 | 4.10 | 3.97 | 4.70 | 4.68 | 3.46 | 3.65 | 3.55 | 3.62 |
| 18 | 6.09 | 6.03 | 6.15 | 5.65 | 5.81 | 5.81 | 5.59 | 5.69 |
| 19 | 8.75 | 8.64 | 8.65 | 8.66 | 8.47 | 8.28 | 8.67 | 8.67 |
| 20 | 3.88 | 3.72 | 3.71 | 3.23 | 3.07 | 3.29 | 2.77 | 3.05 |
| 21 | 6.50 | 5.86 | 7.08 | 7.11 | 6.90 | 6.81 | 6.88 | 6.84 |

Table S2: Lung volumes [L] determined by MRI segmentation algorithm

*Each subject (1-21) underwent 4 MRI sessions (S: session) in weekly intervals each with 2 consecutive MRIs under eDIBH. In total, 168 lung MRIs were contoured and lung volumes calculated.*


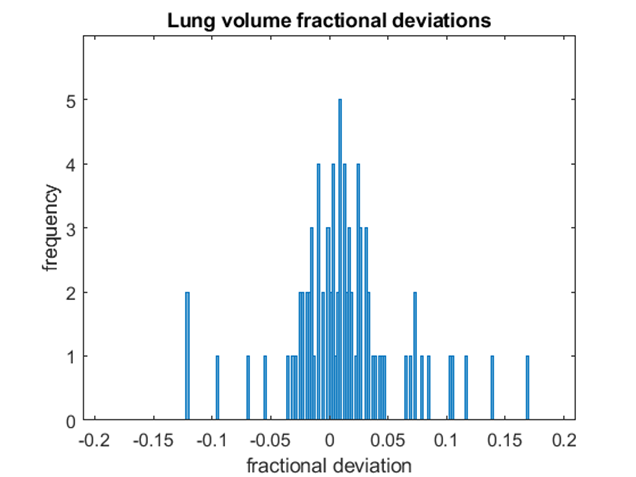


Figure S3: Lung volume fractional deviations

*Four pairs of eDIBH MRI acquisitions were performed within all 4 weekly sessions for 21 subjects yielding 4 deviations from each of the 21 means. The mean of the volume fraction deviations was 0.0167 with a standard deviation of 0.058; 68 of the 84 observations (81%) were within one standard deviation.*


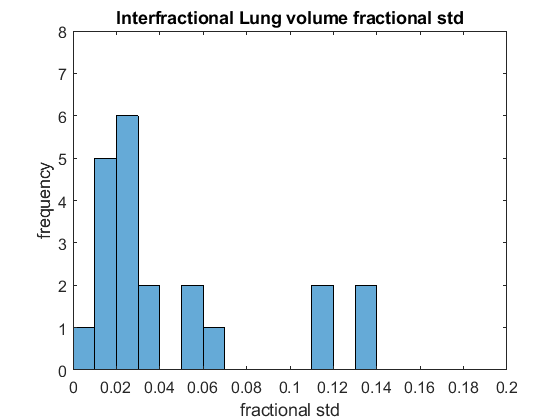


Figure S4: Deviation of session mean about subject grand mean divided by grand mean

*As measure of variation between sessions, the standard deviation of the 4 session means was computed for each subject, yielding 21 standard deviations. The fractional standard deviation was computed as the ratio of standard deviation to mean deviation. 17 of 21 determinations yielded variations of less 7% with median 3%.*


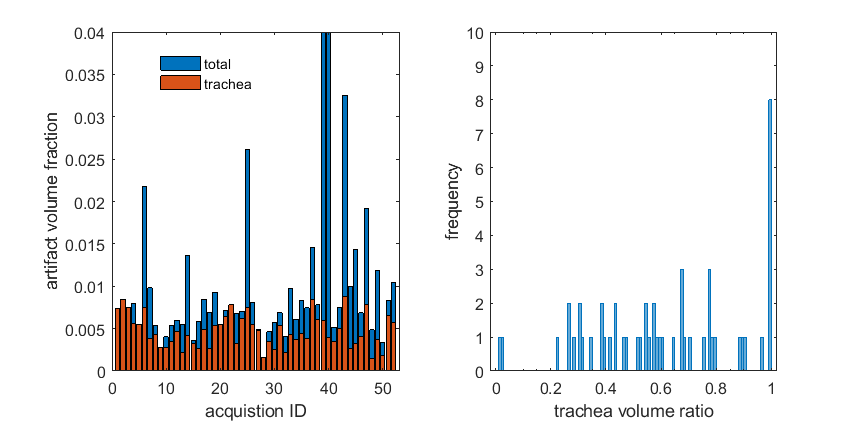


Figure S5: Contribution of trachea to total fractional volumes of artifacts.

*The left panel shows the artifact volume fraction vs the acquisition. The right panel a histogram of the ratio of the trachea contributions to the total.*
